# Effect on Electron Structure and Magneto-Optic Property of Heavy W-Doped Anatase TiO_2_

**DOI:** 10.1371/journal.pone.0122620

**Published:** 2015-05-08

**Authors:** Qingyu Hou, Chunwang Zhao, Shaoqiang Guo, Fei Mao, Yue Zhang

**Affiliations:** 1 College of Science, Inner Mongolia University of Technology, Hohhot, China; 2 College of Arts and Sciences, Shanghai Maritime University, Shanghai, China; 3 School of Material Science and Engineering, Beihang University, Beijing, China; Washington State University, UNITED STATES

## Abstract

The spin or nonspin state of electrons in W-doped anatase TiO_2_ is very difficult to judge experimentally because of characterization method limitations. Hence, the effect on the microscopic mechanism underlying the visible-light effect of W-doped anatase TiO_2_ through the consideration of electronic spin or no-spin states is still unknown. To solve this problem, we establish supercell models of W-doped anatase TiO_2_ at different concentrations, followed by geometry optimization and energy calculation based on the first-principle planewave norm conserving pseudo-potential method of the density functional theory. Calculation results showed that under the condition of nonspin the doping concentration of W becomes heavier, the formation energy becomes greater, and doping becomes more difficult. Meanwhile, the total energy increases, the covalent weakens and ionic bonds strengthens, the stability of the W-doped anatase TiO_2_ decreases, the band gap increases, and the blue-shift becomes more significant with the increase of W doping concentration. However, under the condition of spin, after the band gap correction by the GGA+U method, it is found that the semimetal diluted magnetic semiconductors can be formed by heavy W-doped anatase TiO_2_. Especially, a conduction electron polarizability of as high as near 100% has been found for the first time in high concentration W-doped anatase TiO_2_. It will be able to be a promising new type of dilute magnetic semiconductor. And the heavy W-doped anatase TiO_2_ make the band gap becomes narrower and absorption spectrum red-shift.

## Introduction

In recent years, the stable physical and chemical properties and good photocatalytic performance of anatase TiO_2_ have caused it to attract increased attention in the field of photocatalysis [1–6]. However, given that anatase TiO_2_ is a wide band gap (3.2 eV) semiconductor; its activity can only be shown under ultraviolet light, which should be as short as 380 nm. Ultraviolet light only counts for 5% of the energy of sunlight, whereas visible light accounts for about 45%. Therefore, the improvement of the activity of TiO_2_ under visible light becomes increasingly important.

To realize the visible-light response of TiO_2_, numerous modification experiments have been investigated [1–6]. Individual transition metal doping was found to be one of the effective ways to improve the activity of anatase TiO_2_. Choi et al. [7] studied the effect of 21 kinds of transition metals on the photocatalysis of anatase TiO_2_ and the results showed that Fe^3+^, Mo^5+^, Ru^3+^, Os^3+^, Re^5+^, V^4+^, and Rh^3+^ single doping can improve the visible-light effect of TiO_2_. Yang et al. [8] investigated the effect of W doping on the visible-light effect of anatase and rutile mixed phase, and their results indicated that, when the doping weight fraction percentage of W is within the range of 1.5 wt%–10 wt%, the absorption spectrum of anatase and rutile mixed phase has a red-shift, and when the doping weight fraction percentage is 3wt%, the band gap is the narrowest, the red-shift reaches the most significant level. Through theoretical calculation, Li et al. [9] calculated the effect of W and N single/co-doped on the band gap and absorption spectrum of anatase TiO_2_. The calculated results revealed that the band gap becomes narrower because of W and 2N single/co-doped in anatase TiO_2_ under the situation of spin and the absorption spectrum had a red-shift. Moreover, the red-shift of single-doped W and 2N was more significant than co-doped TiO_2_. Nevertheless, previous reports have only studied the electron spin, rather than the mechanism of magnetic source and narrow gap with theoretical analysis. The two experimental results are opposite. Therefore, to solve the problem, under both conditions of un-spin and spin, we can obtain useful results by simply performing a first-principles study of the electronic structure and magneto-optic property of TiO_2_ heavily doped with W in a doping concentration range similar to previous studied [10, 11].

### Theoretical models and computational method

#### Theoretical models

**Fig 1:**
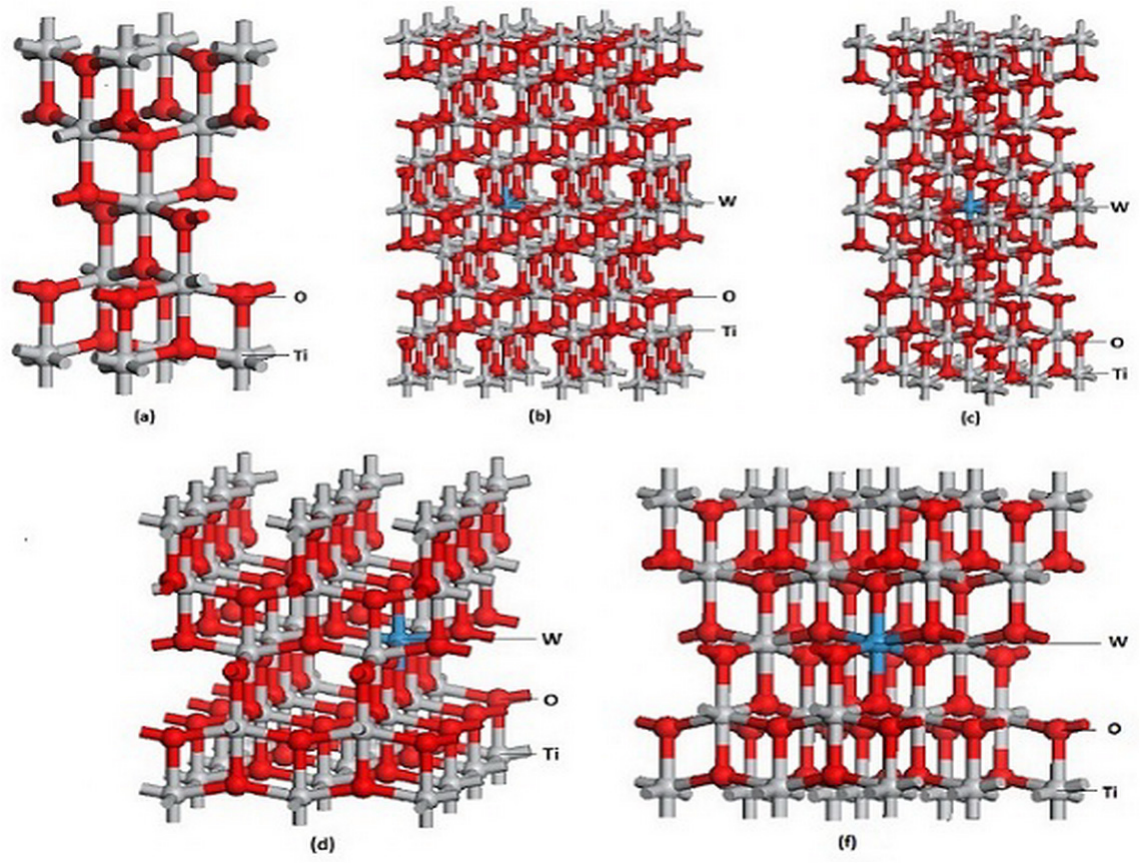
Theoretical models. (A) pure anatase TiO_2_ unit cell, (B) Ti_0.97917_W_0.02083_O_2_ supercell, (C) Ti_0.96875_W_0.03125_O_2_ supercell, (D) Ti_0.95833_W_0.04167_O_2_ supercell, (F) Ti_0.9375_W_0.0625_O_2_ supercell.

Pure anatase TiO_2_ is composed of TiO_6_ octahedra that share edges. Ti^4+^ is the center of octahedron that is constituted by six adjacent O^2−^ ions. Each O atom is surrounded by three Ti atoms that are located in the center of three different octahedra. Each unit cell contains four Ti atoms and eight O atoms. Anatase TiO_2_ has high symmetry of I4_I_/amd. Pure anatase TiO_2_ unit cell and four doping models of Ti_1-*x*_W_*x*_O_2_ (*x* = 0.02083, 0.03125, 0.04167 and 0.0625) were constructed (Fig 1). The mole fractions are 0at%, 0.69at%, 1.04at%, 1.39at% and 2.08at%, respectively. The quality percentages are 0wt%, 4.58 wt%, 6.79 wt%, 8.75wt% and 12.58wt%, respectively.

#### Computational method

All calculations were performed with the Cambridge serial total energy package (CASTEP) [12] (MS5.0) code, which is based on the density functional theory (DFT). Perdew BurkeErnzerhof (PBE) [13] in the scheme of generalized gradient approximation (GGA) is used to treat the exchange-correlation function. Norm conserving pseudo-potential [14] was treated with nonspin polarization. The method of GGA+U was presented to correct the band gap under the condition of electron spin polarization. The value of U (Coulomb interaction energy) was 6 eV for Ti-3d in pure anatase TiO_2_ supercell, whereas the value of U was 4 eV for the W-5d in doped anatase TiO_2_ supercell. They are consistent with the reference [15].To obtain sufficient precision and calculate velocity, the cutoff energy for planewave was assigned to 750 eV during computation. K space lattice point was selected according to Monkhorst-Pack scheme to calculate the integral for total energy and electron density in Brillouin Zone. K-points of Brillouin Zone of pure anatase TiO_2_ supercell were selected for 7×7×3, Ti_0.97917_W_0.02083_O_2_ supercell for 2×3×1, Ti_0.96875_W_0.03125_O_2_ supercell for 3×3×1, Ti_0.95833_W_0.04167_O_2_ supercell for 2×3×3 and Ti_0.9375_W_0.0625_O_2_ supercell for 3×3×3. Planewave energy is converged within 1×10^–5^ eV/atom, where the force on each atom is less than 0.3 eV/nm, the internal stress is below 0.05 GPa, and tolerance deviation is 0.0001 nm. Valence states involved in the calculation process are O (2s^2^2p^4^), W (5s^2^5p^6^5d^4^6s^2^), and Ti (3s^2^3p^6^3d^2^4s^2^).

## Results and Discussion

### Crystal structure, stability and formation energy analysis for un-spin anatase TiO_2_

After geometry optimization, we obtained the lattice parameters, formation energies, and total energies of pure and W-doped anatase TiO_2_. The calculated results are shown in Table 1. According to the results, the optimized geometry lattice parameters of pure anatase TiO_2_ are in good agreement with the experimental values [10]. The results imply that our calculation methods are reliable. According to the principle of the lowest energy in quantum mechanics, one can find when the doping concentration of W is heavier, the total energy increases and the W-doped anatase TiO_2_ become more unstable by the parameters shown in Table 1. Compared with pure anatase TiO_2_, the lattice parameters along the direction of *a*-axis increased and decreased along the direction of *c*-axis [10]. The volume of anatase TiO_2_ is increased. Calculation results are in agreement with the experimental results in reference [10].

**Table 1:** Lattice parameters, total energies, and formation energies of pure and W-doped anatase TiO_2._

Ti_1-*x*_W_*x*_O	*E* (eV)	*E*_f_ (eV)	*a* (nm)	*b* (nm)	*c* (nm)	*V* (nm^3^)
TiO_2_	-9762.61		0.3779	0.3779	0.9657	
Ti_0.97917_W_0.02083_O_2_	-115785.73	4.76	0.3789	0.3789	0.9638	0.0967[^10^]
Ti_0.96875_W_0.03125_O_2_	-76735.25	4.80	0.3791	0.3791	0.9640	0.1385[^10^]
Ti_0.95833_W_0.04167_O_2_	-57209.96	4.87	0.3797	0.3797	0.9625	0.1387[^10^]
Ti_0.9375_W_0.0625_O_2_	-37684.65	4.96	0.3803	0.3803	0.9624	0.1392[^10^]

This table shows some parameters of pure and W-doped anatase TiO_2_.

Recently, the experiment [16] reported that the structure of W-doped anatase TiO_2_ would have a phase transition when the doping concentration is greater than 26.8 wt. %, and if the doping concentration is as heavy as 26.8 wt%, the phase of anatase TiO_2_ would transition to be the rutile. In this work, we study the properties of W-doped anatase TiO_2_ within the doping concentration of 6.79 wt. % to 23.61 wt. % because this range could assures that the doped anatase TiO_2_ was still in the anatase phase and within the scope of heavy doping.

The structure of anatase TiO_2_ phase will be changed (will turn into rutile), and it does not meet the requirements of this paper. Because of the periodicity of the crystal, the heaviest doping concentration we selected in this work was 23.61 wt. %.

We analyzed the relative degree of difficulty of doping anatase TiO_2_ with different ions by comparing impurity formation energies; pure and doping super cells all employ the same size, the impurity formation energy (*E*_*f*_) is defined as follows [17]:

$$E_{f}=E_{TiO_{2}:W}-E_{TiO_{2}}-\mu_{W}+\mu_{Ti}$$ 1

where $E_{TiO_{2}:W}$ and $E_{TiO_{2}}$ are the total energies of W-doped anatase TiO_2_ and pure anatase TiO_2_, respectively. *μ*_*W*_ and *μ*_*Ti*_ are the chemical potentials of W and Ti atom, respectively, which are the energy of one atom in the bulk of W (*Im* $\bar{3}$ *m*) and Ti (*P*6/*mmm*). The calculated results are shown in Table 1. According to the results, we find that the formation energy increases and the doping become more difficult with the increase of W doping concentration, but the difference is not significant.

### Mulliken bond population and bond length analysis of un-spin and spin for W-doped anatase TiO_2_

To analyze the doping mechanism of anatase TiO_2_, the Mulliken bond population and bond length of the W-doped anatase TiO_2_ (Ti_0.95833_W_0.04167_O_2_ and Ti_0.9375_W_0.0625_O_2_) under non-spin and spin conditions have been calculated and shown in Table 2 and Table 3, respectively. According to Table 2, under non-spin condition, the bond lengths of W-O which parallel or vertical to c-axis become longer with the increase of W doping concentration, but the Mulliken bond populations decrease. Meanwhile, the covalent bond weakens, the ionic bond strengthens, and the stability of the W-doped anatase TiO_2_ decreases. These calculation results are in agreement with previous stability analysis. According to Table 3, under spin condition, the bond lengths of W-O which parallel or vertical to c-axis also become longer with the increase of W doping concentration, but the Mulliken bond populations decrease. Comparing to the non-spin W-doped anatase TiO_2_, the bond lengths of W-O in spin W-doped anatase TiO_2_ are longer, the Mulliken bond populations decrease, the covalent bond weakens and the ionic bond strengthens.

**Table 2:** Mulliken bond population and bond length of non-spin Ti_0.95833_W_0.04167_O_2_ and Ti_0.9375_ W_0.0625_O_2_.

model	bond	population	bond length (nm)
Ti_0.95833_W_0.04167_O_2_	W-O (//c)	0.44	0.1958
	W-O (⊥c)	0.47	0.1956
Ti_0.9375_ W_0.0625_O_2_	W-O (//c)	0.43	0.1965
	W-O (⊥c)	0.44	0.1967

This table shows Mulliken bond population and bond length of non-spin Ti_0.95833_W_0.04167_O_2_ and Ti_0.9375_ W_0.0625_O_2._

**Table 3:** Mulliken bond population and bond length of spin Ti_0.95833_W_0.04167_O_2_ and Ti_0.9375_ W_0.0625_O_2_.

model	bond	population	bond length (nm)
Ti_0.95833_W_0.04167_O_2_	W-O (//c)	0.28	0.1965
	W-O (⊥c)	0.34	0.1958
Ti_0.9375_ W_0.0625_O_2_	W-O (//c)	0.26	0.1966
	W-O (⊥c)	0.32	0.1967

This table shows Mulliken bond population and bond length of spin Ti_0.95833_W_0.04167_O_2_ and Ti_0.9375_ W_0.0625_O_2._

### Difference charge density analysis for non-spin and spin W-doped anatase TiO_2_ system

**Fig 2:**
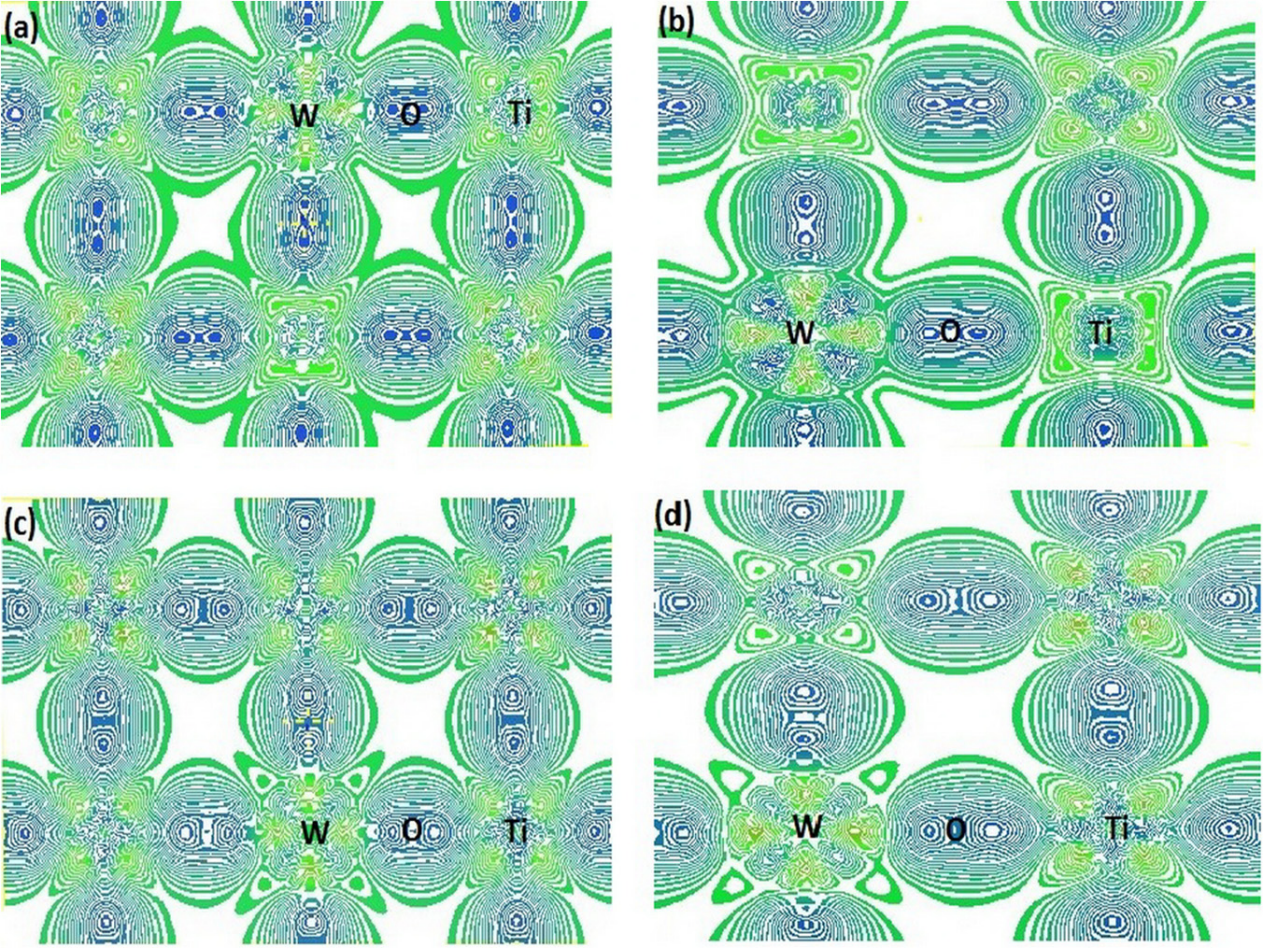
Difference charge density charts of (001) plane of the non-spin and spin for W-doped anatase TiO2. (A) non-spin Ti_0.95833_W_0.04167_O_2_ supercell, (B) non-spin Ti_0.9375_W_0.0625_O_2_ supercell, (C) spin Ti_0.95833_W_0.04167_O_2_ supercell, (D) spin Ti_0.9375_W_0.0625_O_2_ supercell.

To further analyze the interatomic interactions and bonding situation of the W-doped anatase TiO_2_, we calculated the difference charge density of non-spin and spin Ti_0.95833_W_0.04167_O_2_ and Ti_0.9375_W_0.0625_O_2_ and shown in Fig 2. The overlapping extent of the neighboring electronic clouds of W-O in both the non-spin and spin W-doped anatase TiO_2_ weakens with the increase of W doping concentration, which indicates the decrease of ionic bonding and the increase of covalent bonding. Comparing to non-spin W-doped anatase TiO_2_, the overlapping extent of the neighboring electronic clouds of W-O in the spin W-doped anatase TiO_2_ are weaker, and the covalent and ionic bonds weaken and strengthen, respectively. These calculated results are in agreement with those of population and bond length.

### Heavy doping analysis for W-doped anatase TiO_2_

The W doping concentration in Ti_0.97917_W_0.02083_O_2_, Ti_0.96875_W_0.03125_O_2_ and Ti_0.95833_W_0.04167_O_2_ supercells are approximately 6.02×10^20^cm^-3^, 8.71×10^20^cm^-3^ and 1.20×10^21^cm^-3^, respectively, which are greater than the critical concentration of 10^18^ cm^−3^ and are considered as heavy doping. Studies on band structures of W-doped anatase TiO_2_ are necessary to support this possibility.

### Band gap analysis for un-spin W-doped anatase TiO_2_

**Fig 3:**
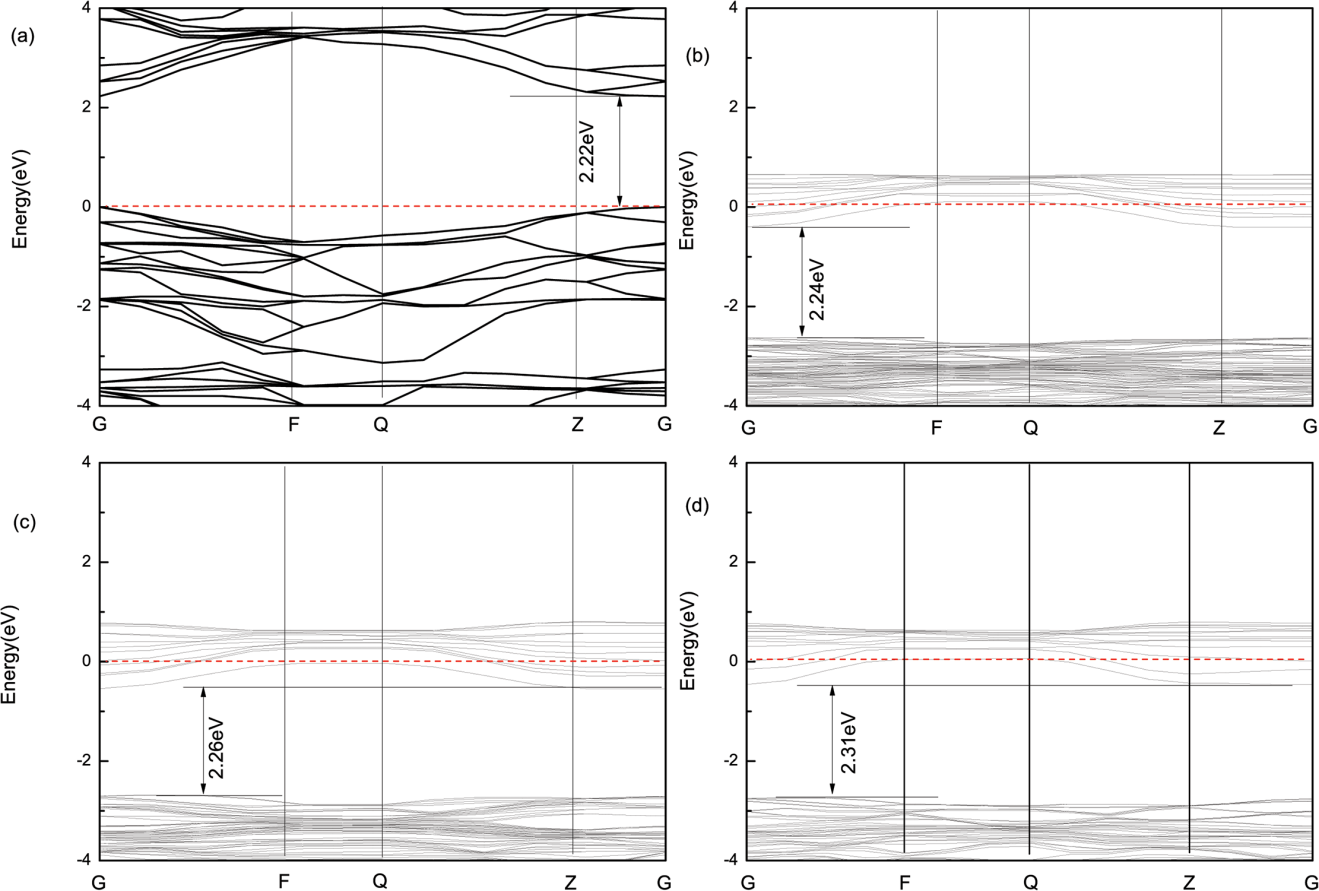
Band structure for pure and W-doped anatase TiO_2_. (A) pure anatase TiO_2_ unit cell, (B) Ti_0.97917_W_0.02083_O_2_ supercell, (C) Ti_0.96875_W_0.03125_O_2_ supercell, (D) Ti_0.95833_W_0.04167_O_2_ supercell.

Under the condition of non-spin, Fig 3A–3D are band structures of pure and W-doped anatase TiO_2_ (in this paper, all the Fermi levels have been specified to be 0 eV). As shown in Fig 2A, the band gap (*E*_g_) of pure anatase TiO_2_ is 2.22 eV, which is in agreement with the previously reported value [18, 19]. However, this band gap value is less than the experimental value of 3.2 eV. For oxide, this is a common phenomenon, which is produced by using the GGA approximation method [20] under the condition of nonspin. This discrepancy does not have an impact on the analysis of the relative values of the calculated results of pure vs. doped TiO_2_. The band structures of supercell Ti_0.96875_W_0.03125_O_2_, Ti_0.9375_W_0.0625_O_2_, and Ti_0.875_W_0.125_O_2_ are shown in Fig 3B, Fig 3C, and Fig 3D, respectively. The Fermi level of both doping models observably shifts upward into the conduction (CB), which is in agreement with the heavy doping analysis. Meanwhile, the band gap of Ti_0.97917_W_0.02083_O_2_, Ti_0.96875_W_0.03125_O_2_ and Ti_0.95833_W_0.04167_O_2_ super cells are 2.24 eV, 2.26 eV, and 2.31 eV, respectively. The calculated results are in agreement with the experimental results [10]. Subsequently, we will discuss the broadened mechanism of the band gap for W-doped anatase TiO_2_ from the point of renormalization theory and partial density of states (PDOS) under the condition of non-spin.

### Renormalization theory analysis

We know from renormalization theory that two reasons exist for the broadening of band gap for Ti_0.97917_W_0.02083_O_2_, Ti_0.96875_W_0.03125_O_2_ and Ti_0.95833_W_0.04167_O_2_ under the condition of non-spin. On the one hand is the so-called Burstein-Moss effect produced by heavy W doping in TiO_2_, which makes the optical absorption edge shift toward the low-energy region and band gap broaden. On the other hand is the band gap narrowing produced by the band gap renormalization effect from the interaction between electric charge or the overlap between the impurity and defect bands [21]. The calculated results in this work show that the former one is greater than the latter one, resulting in the broadening of the band gap, and under the condition of non-spin, when the W concentration is heavier, the band gap becomes broader.

### Partial density of states analysis for un-spin W-doped anatase TiO_2_

**Fig 4:**
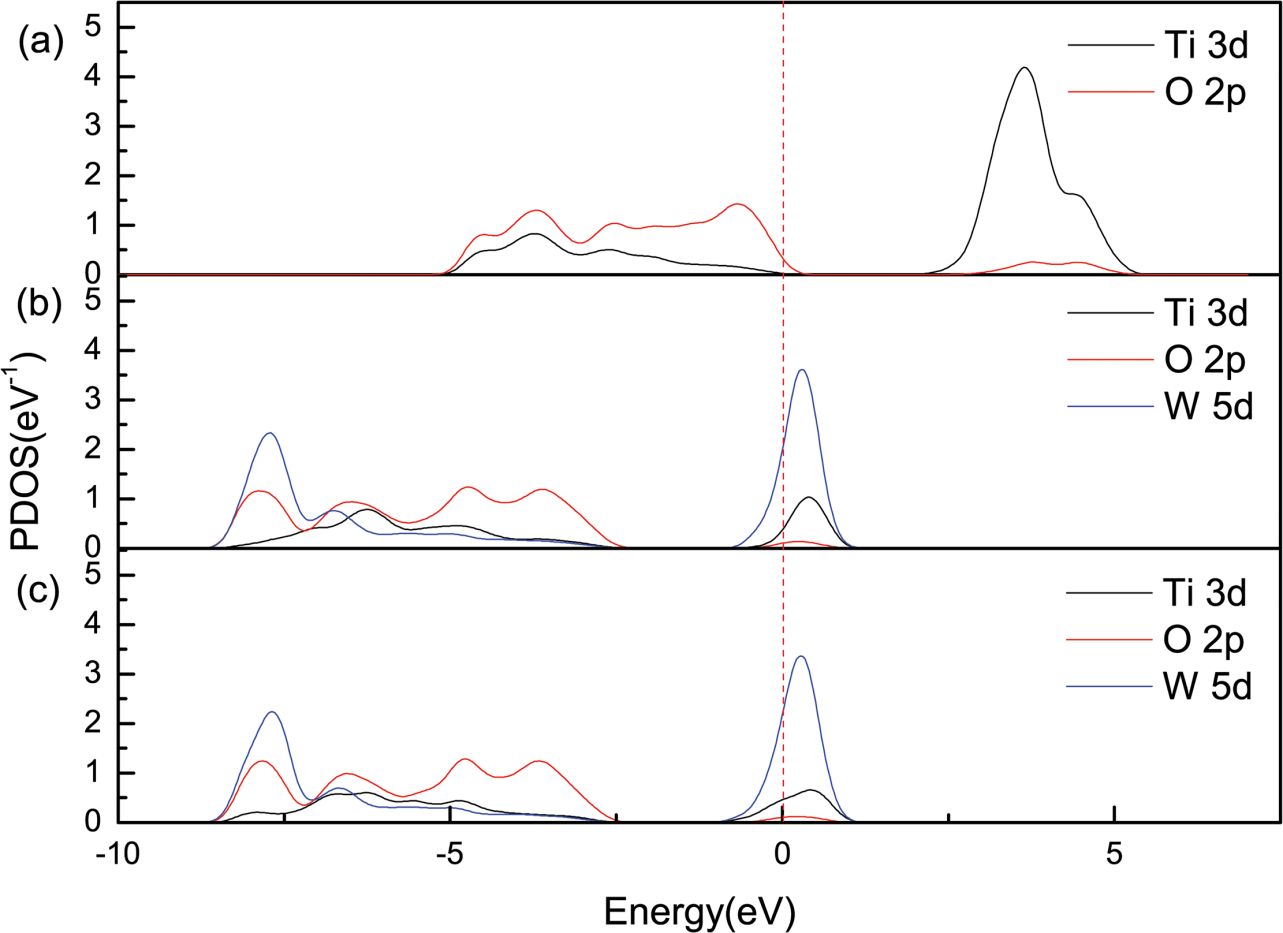
PDOS for pure and W-doped anatase TiO_2_. (A) pure anatase TiO_2_ unit cell, (B) Ti_0.97917_W_0.02083_O_2_ supercell, (C) Ti_0.96875_W_0.03125_O_2_ supercell.

The PDOS of pure anataseTiO_2_ and of the doping systems are shown in Fig 4A, Fig 4B, and Fig 4C. From Fig 4A, we can see that the pure anatase TiO_2_ band gap is formed by the anti-bonding-like state of the d orbital and the bonding-like state of the p orbital, both of which are formed by the interaction of the Ti-3d states and the O-2p states. We can see from Fig 4B and Fig 4C that W-5d states have lower energy than Ti-3d states, and that the conduction band minimum (CBM) is formed by the W-5d states, resulting in the downward shift of CB. Meanwhile, Ti-3d states and W-5d states become stronger; the interaction between d-d states also become stronger and the downward shift of CB becomes more obvious with the increase of W doping concentration. Due to heavy doping, the Fermi level of both doping models shifts upward into the CB; this results in the appearance of the Urbach band-tail effect. This is in agreement with the band structure and heavy doping analyses.

The size variation of Fermi energy level in pure and W-doped anatase TiO_2_ also explains that the band gaps of doped system broaden with the increase of W doping concentration. We calculated and obtained Fermi energy level in pure anatase TiO_2_ which is approximately 4.30eV. However, the Fermi energy levels of Ti_0.97917_W_0.02083_O_2_ and Ti_0.96875_W_0.03125_O_2_ supercells are approximately 5.48eV and 5.49eV, respectively. By increasing the W doping concentration, the following results were obtained: the Fermi energy level of W-doped anatase TiO_2_ increases and is higher than that of pure anatase TiO_2_. According to the semiconductor theory, the higher Fermi energy level is, the more carriers filling conduction band is and the stronger Burstein-Moss effect will be. Therefore, the band gaps of W-doped anatase TiO_2_ broaden with the increase of W doping concentration. This coincides with the experimental results [10].

The VB shifts toward the low-energy region due to the existence of the interaction of the d-d states and the repulsion effect of the p-d states in the tetrahedron structure. The interaction of the d-d states causes VB to shift toward the low-energy region, whereas the interaction of the p-d states causes VB to shift toward the high-energy region. Fig 4B and Fig 4C show the PDOS of W-doped anatase TiO_2_, illustrating that when the doping concentration of W becomes heavier, the Ti-3d states become stronger and the interaction of the d-d states becomes stronger than the repulsion effect of the p–d states. Therefore, with the increase in W doping, the VB shift toward the low-energy region becomes more significant.

In summary, under the condition of non-spin, the broadening of the band gaps are caused by the CB shift toward the low-energy region less than the VB for Ti_0.97917_W_0.02083_O_2_ and Ti_0.96875_W_0.03125_O_2_, compared with pure anatase TiO_2_. Moreover, when the W doping is heavier, the CB shift becomes less than the VB because of the greater broadening of band gap, and this is in accordance with the analysis of the renormalization theory and with the experimental results [10].

### Absorption spectrum analysis for un-spin pure and W-doped anatase TiO_2_

It is well known that the optical properties of the medium can be described by complex dielectric response function *ε*(*ω*) = *ε*_1_(*ω*) + *iε*_2_(*ω*) in leaner response range, in which *ε*_1_ = *n* ^2^−*k* ^2^ and *ε*_2_ = 2*nk*. The real part *ε*_1_(*ω*) and imaginary part *ε*_2_(*ω*) can be calculated according to the dispersion relation of Kramers–Kronig. In this way, absorption coefficient *α*(*ω*), can be obtained. Omitting the derivation process, the concerned formulas can be written as:

$$\varepsilon_{2}(\omega )=\frac{c}{\omega^{2}}\sum_{V,C}\int_{BZ}\frac{2}{{\left(2\pi \right)}^{3}}{\left|M_{CV}(k)\right|}^{2}\cdot \delta (E_{C}^{k}-E_{V}^{k}-\hbar \omega )d^{3}k$$ 2

$$\varepsilon_{1}(\omega )=1=\frac{2}{\pi }\rho_{0}\int_{0}^{\infty }\frac{\omega '\varepsilon_{2}(\omega )}{\omega {'}^{2}-\omega^{2}}d\omega$$ 3

α(ω)=2[ε12(ω)+ε22(ω)−ε1(ω)]12 4

where, subscript *C* and *V* represent the conduction band and the valence band, respectively, *BZ* is the first Brillouin zone, *k* is the reciprocal lattice vector,|**M**_*CV*_(*k*)|^2^is the momentum matrix element, *c* is a constant, *ω* is the angular frequency, $E_{C}^{k}$ and $E_{V}^{k}$ is the intrinsic energy level. The above formulas provide a theoretical foundation for analyzing the band structure and the optical properties of a crystal.

**Fig 5:**
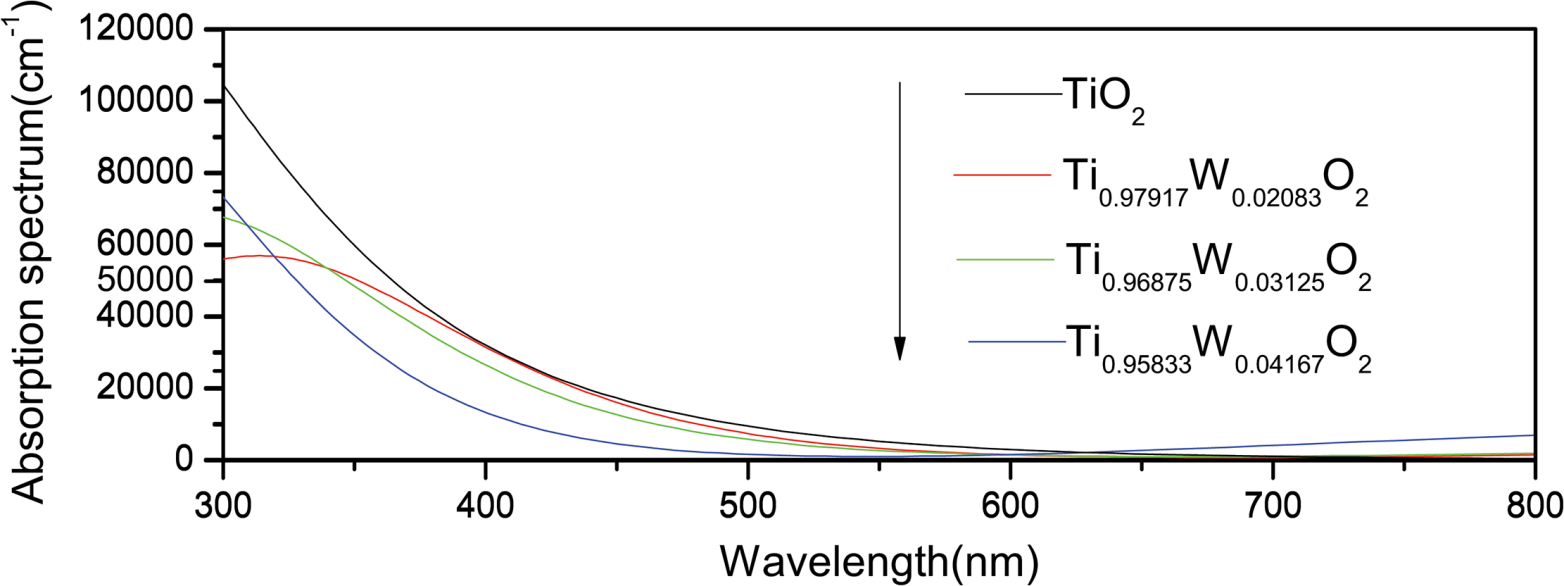
Optical absorption curves for pure and W-doped anatase TiO_2_ under the condition of un-spin.

Under the condition of nonspin, the optical absorption curves for Ti_1-x_W_x_O_2_ (x = 0, 0.02083, 0.03125 and 0.04167) supercells are shown in Fig 5. According to this Fig., for wavelength ranging from 300nm to 800nm, the absorption spectrum has a blue-shift in the visible-light region when a Ti atom is replaced by a W atom, which is in agreement with the experimental results [10, 20]. When the doping concentration of W becomes heavier, the blue-shift of the absorption spectrum becomes more significant. This is not conducive to the doping system to be converted into visible-light effect.

### Band gap and magnetic analysis of spin W-doped anatase TiO_2_

**Fig 6:**
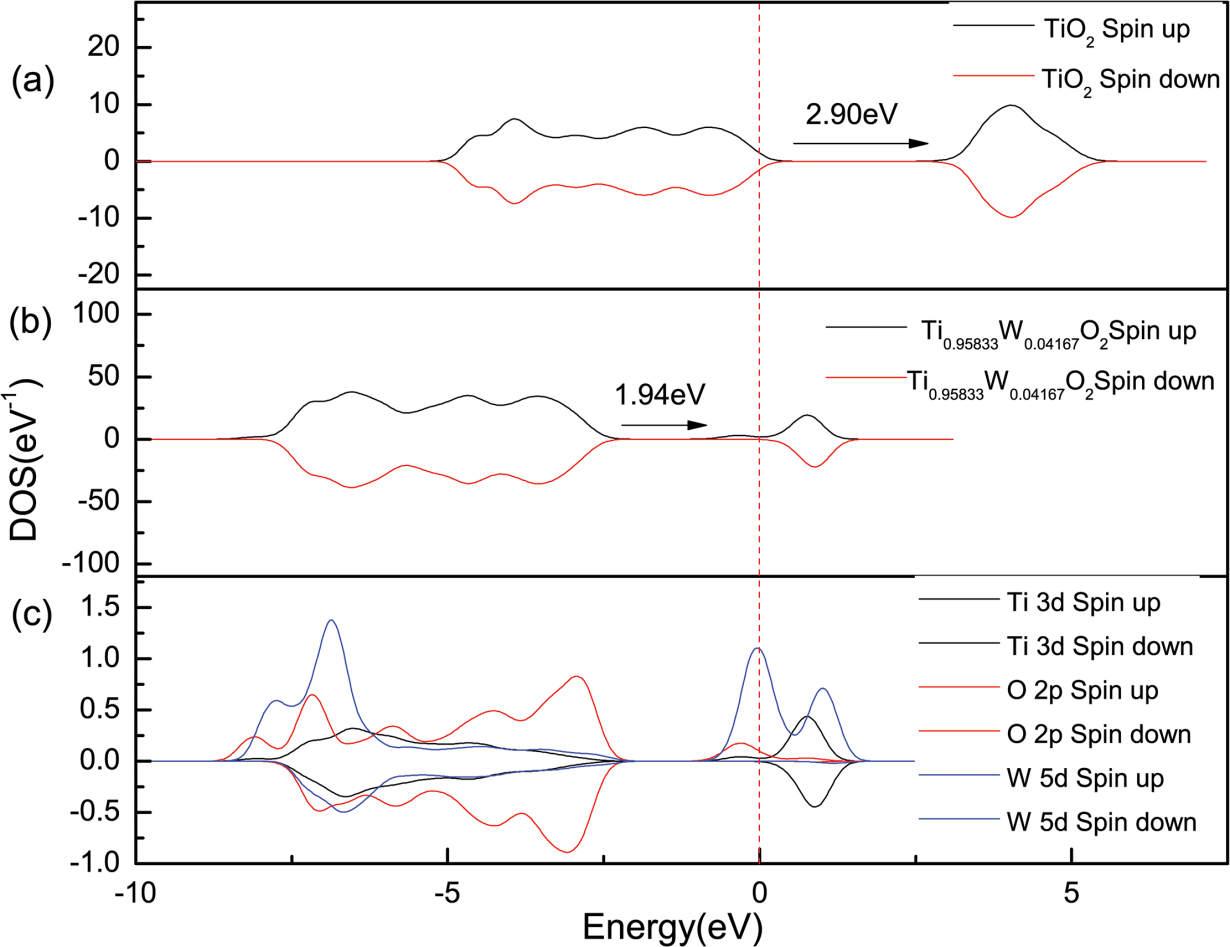
Density of states for pure and W-doped anatase TiO_2_ under the condition of spin. (A) Total density of state for pure anatase TiO_2_ unit cell, (B) Total density of state for Ti_0.95833_W_0.04167_O_2_ supercell, (C) Partial density of state for Ti_0.95833_W_0.04167_O_2_ supercell.

Under the condition of un-spin, the method of GGA+U was also presented to correct the band gap. The calculated total density of state of pure anatase TiO_2_ is shown in Fig 6A, and the band gap is 3.0 eV for pure anatase TiO_2_. The value is close to the experimental value of 3.2 eV [7], which shows that the method of GGA+U is reasonable.

The total density of state (TDOS) and partial density of state (PDOS) of Ti_0.95833_W_0.04167_O_2_ supercell were calculated under the condition of spin, as shown in Fig 6B and Fig 6C, respectively. The band gap widths are 1.94eV from Fig 6B. Calculations indicate that the doping amount is large and that the width of band gap is narrowing. Meanwhile, we can see that the electron numbers of up-spin and down-spin are obviously not the same. This shows the magnetism of the doping systems; Moreover, the Fermi surface gets into the up-spin conduction band and it does not get into the down-spin conduction band, the doping system forms half metal dilute magnetic semiconductor. Comparing with the traditional dilute magnetic semiconductor (DMS), W doping does not bring the problem of sediment due to W have no magnetic. Hence, it is the first time to find that high concentration of W-doped anatase TiO_2_ has a conduction electron polarizability of as high as near 100%. It will be a very promising new type of DMS. From Fig 6C, we can see that when a Ti atom becomes replaced by a W, the electronic exchange interaction occurred for doped TiO_2_, that is $(e_{cb}^{-})+W^{6+}\to W^{5+}$ and $W^{5+}+O_{2}\to W^{6+}+O_{2}^{\cdot -}$ [22]. This phenomenon leads to the electron, which is overlapped by Ti-3d and W-5d state, to shift to the O-2p state, causing the CB to shift toward the low energy region and the band gap to narrow. To further explain in theory, this phenomenon occurs because the 5d orbitals of W is discontent; with the W doping, the different spin electronics were redistributed while the electronic distribution and bonding was formed, and this is shown as the electrons located in the similar level becoming divided due to the effect of the molecular field. Hence, the spin up and spin down electrons become separated in the energy scale, and this is the so-called spin splitting. However, the splitting process was a spontaneous energy decrease. Therefore, the CB shifts downward more than the VB, which caused the narrowing band gap of anatase TiO_2_.

The source of the magnetism of the doping system is mainly in the Fermi surface. From Fig 6C, we can see that the O-2p states have no contribution to the magnetism. The magnetism of the system arises from the overlap between the Ti 3d orbitals and the W 5d orbitals, as well as the electron-electron exchange interactions. This is consistent with the theoretical explanation wherein the electronic structure of ZnCoO system with the oxygen vacancy was calculated using first-principles the plate-wave ultra-soft pseudopotential method by Ihm et al. [23]. The calculated total magnetic moment is 1.99 *μ*_*B*_, and *μ*_*B*_ is the Bohr magneton. This is consistent with the result wherein reported [24]. It is also consistent with the experimental result wherein the magnetism arises through a similar non-magnetic transition metal V or Cu single-doped anatase TiO_2_ [25, 26].

### Absorption spectrum analysis for pure and spin W-doped anatase TiO_2_

**Fig 7:**
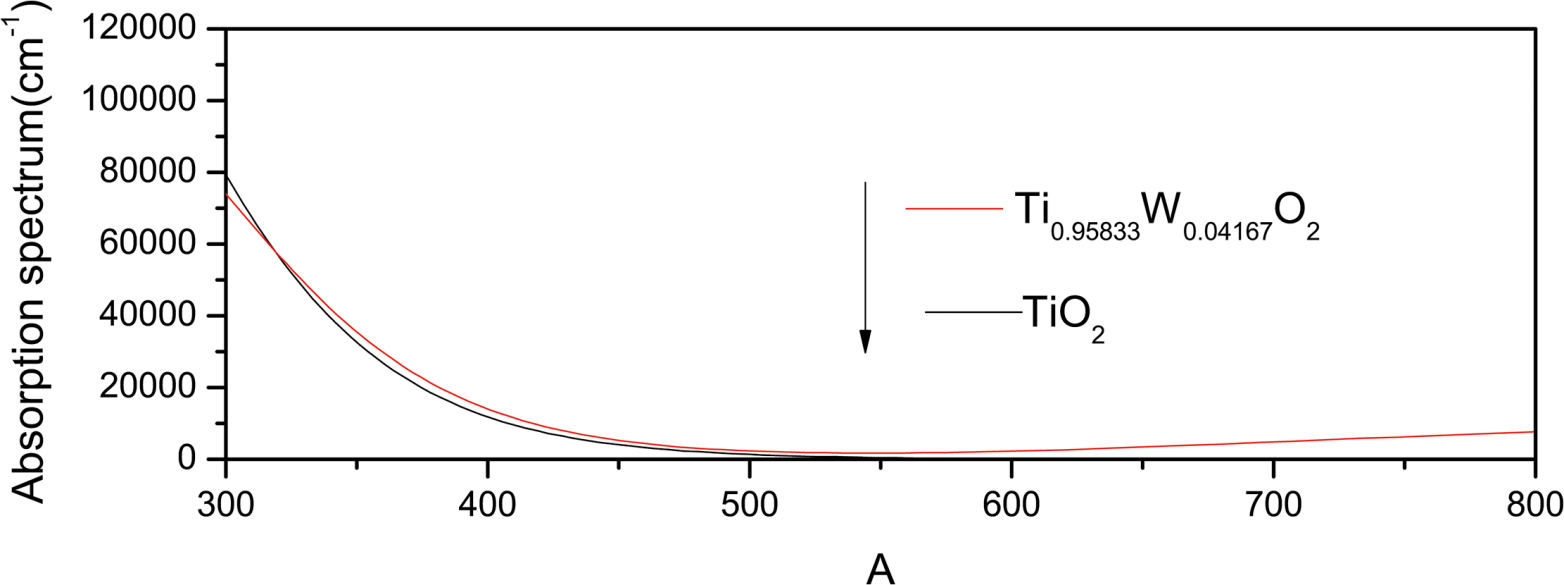
Optical absorption curves for pure and W-doped anatase TiO_2_ under the condition of spin.

Under the condition of spin, the optical absorption curves for pure and W-doped anatase TiO_2_ are shown in Fig 7. For wavelength ranging from 300 to 800nm, we can see that absorption spectrum has a red-shift in the visible-light region in doping supercells Ti_1-*x*_W_*x*_O_2_ (*x* = 0.0625), which is in agreement with the experimental results [11, 27, 28, 29]. This would provide certain theory guidance for the design and preparation of the photocatalyst.

### Analysis of existing problems

Studies on the absorption spectrum of W-doped anatase TiO_2_ systems under similar concentration have been previously experimental reported [10, 11]; however, the results were opposite. The space dimension of doping system is in a range of 70–980nm in reference [10], the doping system characterized by macro scale effect mainly. However, in another report [11] the space dimension of doping system is in a range of 8–15nm. The spatial scale of doping system down to the near atomic scale and then the quantum effect cannot be neglected which leads to a magneto-optic property of the nanoparticles and the macro scale effect are obviously different. This is in agreement with the nanoscale quantum effects in low range wherein experimental results [27]. In the same way, when the space dimension of doping system is in the range of 13.3–25.1nm [28], the experimental results showed that a red-shift absorption spectra of anatase TiO_2_ in similar doping concentration range. It can also support this viewpoint. So, we propose that in order to control the optical properties of W-doped anatase TiO_2_, the scale of doped system and doping concentration of W are all crucial factors. For increasing the visible effect of the W-doped anatase TiO_2_, a high concentration of W and a near atomic scale system should be realized.

### Conclusions

In this work, we applied both the conditions of spin and un-spin and the effect of heavy W doping on the electronic structure and absorption spectrum of anatase TiO_2_ have been studied from first principles. The conclusions could be summarized as follows:

Under the condition of un-spin, compared to pure anatase TiO_2_, when the doping concentration is heavier, the lattice parameters parallel to the direction of the *a*-axis increases and those parallel to the direction of *c*-axis decreases. The equivalent volumes of crystal cells of doping models are greater than the unit cell of pure, and the formation energy increases. The doping becomes more difficult, but the difference is not significant. The Mulliken bond population of W-O in the W-doped anatase TiO_2_ decreases with the increase of W doping concentration. Meanwhile, the covalent bond weakens, the ionic bond strengthens, the total energy increases, and the doping system becomes more unstable. And the band gap becomes broader; the blue-shift of the absorption spectrum becomes more significant.

Under the condition of spin, it is the first time to find that the semimetal diluted magnetic semiconductors can be formed by heavy W-doped anatase TiO_2_, high concentration of W-doped anatase TiO_2_ has a conduction electron polarizability of as high as near 100%. It will be a very promising new type of dilute magnetic semiconductors. The doping system of TiO_2_ becomes magnetic, and when the doping concentration of W is higher, the band gap becomes narrower and the red-shift of the absorption spectrum becomes more significant. The calculation results coincide with the experimental results [11, 27, 28, 29]. These results provide a theory reference for designing the new type of photo catalyst.
